# Critical-sized bone defects: essential concepts for the higher orthopaedic examination

**DOI:** 10.1007/s00590-026-04999-1

**Published:** 2026-10-22

**Authors:** Katie Hutchinson, Maria Vitenberg, Pramin Raut, Alex Trompeter

**Affiliations:** 1 St George’s University Hospitals NHS Foundation Trust, London, UK; 2 City St George’s, University of London, London, UK

**Keywords:** Bone defects, Bone loss, Fracture-related infection, Distraction osteogenesis, Non-union

## Abstract

**Purpose:**

To provide a practical review of the assessment and management of critical-sized bone defects (CSDs) for candidates preparing for higher orthopaedic examinations.

**Methods:**

This narrative review considers definitions and classification, clinical assessment, acute management, reconstructive techniques and salvage options.

**Results:**

A critical-sized bone defect (CSD) is unlikely to heal without intervention because of its size and location, although no numerical threshold defines criticality. Assessment should consider skeletal stability, infection, vascularity, soft tissues, host factors and likely function. After resuscitation, priorities include debridement, skeletal stabilisation and soft-tissue cover. Definitive bone loss should be assessed after debridement, which may require multiple operations. Reconstruction may be single-stage or staged. Options include bone grafting or acute shortening in selected cases, bone transport, the induced-membrane technique and vascularised bone transfer. Patient-specific implants show promise, but long-term evidence is limited. Planning should involve an orthoplastic team, with microbiology input if infection is suspected. Amputation should be considered if reconstruction is unlikely to yield a functional limb.

**Conclusion:**

Successful management requires infection control, stable fixation and reliable soft-tissue cover. The reconstructive approach should reflect the defect and the patient’s circumstances, balancing likely functional benefit against the burden of treatment.

## Introduction

Critical-sized bone defects (CSD) remain one of the most demanding problems encountered in orthopaedic trauma and limb reconstruction [1]. Their management requires a comprehensive understanding of bone biology, biomechanics, soft-tissue reconstruction, and musculoskeletal infection, as well as familiarity with a range of complex reconstructive techniques.

Segmental bone loss most commonly follows high-energy trauma, fracture-related infection, tumour resection or failed previous reconstructive procedures. Such injuries are associated with prolonged treatment, multiple surgical interventions and substantial patient morbidity. Although no universally accepted definition exists, segmental bone loss exceeding 2 cm in length and involving more than 50% of the cortical circumference is widely considered a critical defect. The United States Food and Drug Administration (FDA) recognises the American Society for Testing and Materials (ASTM) definition of a critical-sized segmental defect as one that will not fill with viable bone without treatment; however, this definition provides no clinically applicable dimensional threshold [2]. The OTA Open Fracture Classification grades bone loss from no loss to segmental loss (B1–B3), but neither quantifies the defect nor defines criticality [3]. Because size alone does not reliably predict healing, a critical defect is best understood as one that compromises the skeleton’s structural or functional integrity and is unlikely to heal without intervention. This judgement also depends on defect morphology, anatomical location, mechanical stability, vascularity, soft-tissue injury, infection and host biology.

Advances in limb reconstruction have led to the development of multiple effective reconstructive techniques, including acute shortening and relengthening, bone transport, the induced membrane technique (Masquelet), bone grafting, and augmentation with three-dimensional (3D)-printed implants. However, no single method is universally applicable, and successful treatment depends upon careful assessment of the osseous defect, local and systemic risk factors, soft-tissue status, and the presence of infection. It should be recognised that management comprises distinct acute and definitive phases. In the acute setting, the priority following resuscitation is debridement of devitalised, contaminated, and infected tissue, dead space management, skeletal stabilisation, and soft-tissue management. The full extent of bone loss may become clear only after debridement is complete, sometimes over more than one operation; definitive reconstruction can then be planned [1].

This review provides a structured overview of the assessment and management of critical-sized bone defects, encompassing both the principles of acute management and contemporary reconstructive techniques. Emphasis is placed on evidence-based practice, practical decision-making, and concepts of particular relevance to higher surgical trainees and candidates preparing for board examinations.

### Classification

Bone defects may be classified broadly as critical or non-critical and, according to their extent, as incomplete or complete segmental defects. Their anatomical location may be described separately as diaphyseal/intercalary, metaphyseal/periarticular or articular. This distinction is a useful starting point, though management is determined by the size and anatomical location of the defect, the state of the soft-tissue envelope, mechanical stability, host factors and the local biological environment.

Several established classifications describe the wider injury without specifically characterising the osseous defect itself. The Gustilo–Anderson, Orthopaedic Trauma Society and OTA Open Fracture classifications grade the severity of open fractures and their associated soft-tissue injury, while the AO/OTA Fracture and Dislocation Classification is concerned principally with fracture location and morphology [4]. The Ganga Hospital Open Injury Severity Score includes bone loss as one element within a broader prognostic assessment, alongside soft-tissue injury and host factors [5]. These systems are of considerable value in prognosis and initial management but do not offer a comprehensive classification of defect morphology or criticality, and are best regarded as complementary to defect-specific classifications rather than a substitute for them.

The Ferreira–Tanwar classification was devised specifically as a treatment-orientated system for post-traumatic, critical-sized segmental diaphyseal long-bone defects [6]. Defects are graded by size (Type I, <2 cm; Type II, 2–6 cm; Type III, 6–12 cm; Type IV, >12 cm), by soft-tissue status (α, intact; β, reconstructable; γ, non-reconstructable) and by host status (A, healthy; B, compromised; C, unsuitable for reconstruction). Taken together, this provides a practical basis on which to select an appropriate reconstructive strategy.

The Universal Long Bone Defect Classification, by contrast, follows an AO/OTA-style approach in standardising the description of long-bone defects according to anatomical location and morphological characteristics [7]. Between them, these defect-specific systems allow for more precise characterisation of the osseous defect itself, while the established open-fracture and injury-severity classifications continue to serve their purpose in describing the accompanying soft-tissue injury, host status and overall prognosis.

### Clinical assessment

The history should establish the mechanism and timing of injury, previous surgical interventions, and any evidence of infection, including wound breakdown, persistent discharge, or previous microbiological diagnoses. Co-morbidities known to impair bone healing, such as diabetes mellitus, peripheral vascular disease, smoking, immunosuppression, and nutritional deficiency, should be identified [8, 9]. The patient's pre-injury function, current symptoms, and treatment expectations should also be explored.

Patients presenting for reconstruction will frequently have undergone initial debridement and provisional stabilisation, typically with an external fixator. Clinical examination should therefore assess the condition of pin sites, frame stability, and the relationship of the construct to planned reconstructive options [10, 11]. Particular attention should be paid to the quality of the soft-tissue envelope, including previous incisions, soft-tissue loss, flap viability or the requirement for flap reconstruction, evidence of persistent infection such as a sinus tract. Neurovascular status, adjacent joint function, and musculotendinous integrity should also be assessed, as these are key determinants of reconstructive strategy. Limb alignment and limb-length discrepancy are more accurately assessed radiographically during pre-operative planning, except in cases managed with acute shortening where restoration of length forms part of the reconstructive strategy [12].

### Investigations

Plain radiographs (XR) are the initial investigation of choice and the use of an appropriately scaled markers can aid accurate measurement of the defect. Computed tomography (CT) may further characterise defect morphology, comminution, and articular involvement. Magnetic resonance imaging (MRI) and, in selected cases, 18F-FDG PET-CT may be useful for evaluating osteomyelitis, occult infection, and the extent of soft-tissue involvement [13]. Laboratory investigations should include inflammatory markers and assessment of modifiable host factors. Where infection is suspected, definitive diagnosis relies on multiple deep tissue cultures obtained at surgical debridement [14].

### Management principles

The aim of treatment is to achieve an infection-free, mechanically stable limb with soft-tissue coverage and the best attainable function. The bone defect should be considered within the wider injury, including contamination, vascularity, soft-tissue and neuromuscular damage, host factors, and patient expectations.

Initial management comprises thorough debridement, preservation of viable bone and periosteum, antimicrobial therapy, skeletal stabilisation, and early soft-tissue coverage. The defect should be assessed following definitive debridement, when its true dimensions and biological characteristics are apparent [15, 16].

Management may be single-stage or staged. Single-stage reconstruction is appropriate when adequate debridement, stable fixation, defect reconstruction, and reliable soft-tissue coverage can be achieved safely.

**Fig. 1:**
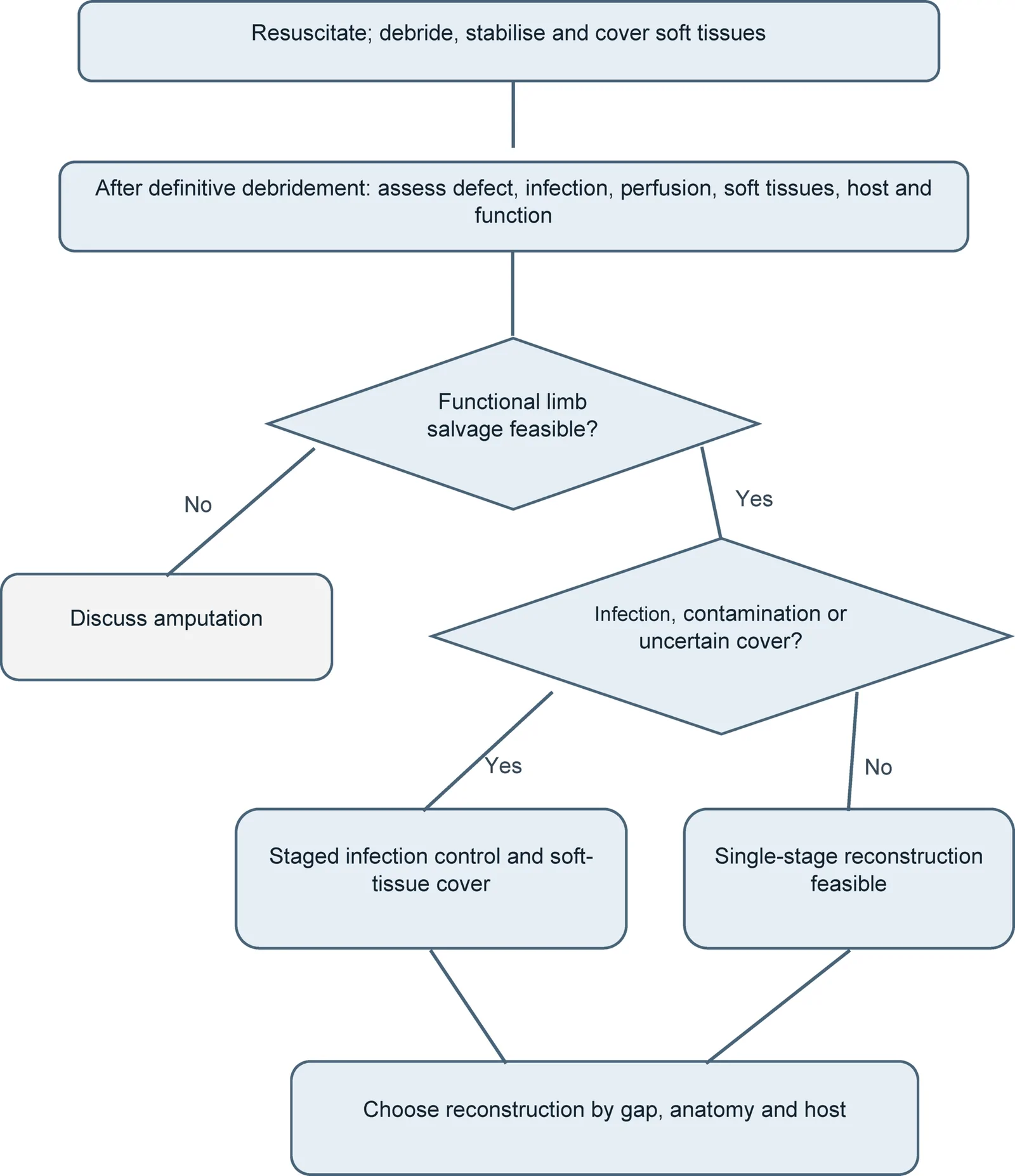
Flowchart for critical-sized bone defect reconstruction

Staged treatment is often preferred in the presence of infection, substantial contamination, uncertain tissue viability, or inadequate soft-tissue cover. In these circumstances, debridement, skeletal stabilisation, and soft-tissue coverage take priority before definitive defect reconstruction. Each phase may require more than one operation. Treatment should be planned within an orthoplastic multidisciplinary team, with microbiology input when infection is suspected or confirmed, considering the anticipated functional benefit, reconstructive burden, complications, and rehabilitation. Where a durable functional limb is unlikely, amputation should be considered a reconstructive option rather than treatment failure. Figure 1 summarises the proposed pathway from post-debridement assessment to single-stage or staged reconstruction and selection of the osseous technique.

### Single-stage reconstruction

Single-stage reconstruction aims to achieve definitive skeletal stability, defect management, and soft-tissue coverage during the initial treatment episode. It is appropriate only in carefully selected patients with a favourable biological environment and may comprise acute grafting, reimplantation of an extruded segment, or acute shortening [17].

### Acute grafting

Immediate graft reconstruction may be considered after adequate debridement when stable fixation and reliable soft-tissue coverage can be achieved. A limited defect may be treated with internal fixation and autologous bone graft, whereas a vascularised fibular graft may be considered for a larger segmental defect [18]. Substantial contamination, devitalised tissue or uncertain soft-tissue coverage favours staged reconstruction. The risk of deep infection must be balanced against the potential benefit of early reconstruction.

### Reimplantation of extruded bone

Reimplantation of an extruded bone segment may be considered where the fragment is structurally suitable and adequate decontamination, stable fixation and viable soft-tissue coverage can be achieved. Reimplantation may be particularly valuable for fragments containing an articular surface, such as the talus or a femoral condyle: missing diaphyseal bone can be reconstructed, whereas native joint cartilage is difficult to replace. Preparation generally comprises mechanical removal of debris, copious saline irrigation and immersion in an antiseptic or antibiotic solution; autoclaving, gamma irradiation and temporary subcutaneous storage have also been described, although no method has demonstrated superiority. The de-vascularised fragment provides an anatomically matched autologous scaffold but may require supplementary bone grafting. Evidence is limited to case reports and small case series, and infection, resorption, non-union and further surgery remain important risks [19–22].

### Acute shortening

Acute shortening can eliminate or reduce the defect, improve bone contact, and facilitate tension-free soft-tissue closure. It is particularly useful for smaller defects where shortening will not cause unacceptable limb-length discrepancy, neurovascular compromise, or functional impairment [23, 24]. It may provide a pragmatic definitive solution in selected older or low-demand patients, including those with severe open ankle injuries**.** Acute shortening may also form the first phase of a staged reconstruction, followed by re-lengthening using distraction osteogenesis.

### Staged reconstruction

Staged reconstruction is generally preferred where there is contamination, infection, uncertain tissue viability, or inadequate soft-tissue coverage. The initial stage aims to eradicate infection, excise all non-viable tissue, restore stability, manage dead space, and obtain durable soft-tissue coverage. Local antibiotic carriers may deliver high antimicrobial concentrations whilst maintaining the defect for later reconstruction [16, 25].

Progression to bone regeneration requires evidence that the limb is clinically quiescent. The wound should be healed and dry, without erythema, drainage, or recurrent breakdown. Inflammatory markers may remain elevated after surgery and should be interpreted in context rather than used as an isolated threshold. Serial radiographs should show no progressive osteolysis, implant loosening, or bone resorption. No single investigation confirms eradication of infection; the decision should be based on the combined clinical, biochemical, radiological, and microbiological findings [14].

The following techniques form part of a staged approach to reconstruction, with initial debridement, stabilisation and soft-tissue management followed by restoration of bone continuity. The sequence and number of procedures depend on the technique and the condition of the limb.

### Distraction osteogenesis

Distraction osteogenesis, based on the principles described by Ilizarov, permits reconstruction of critical-sized segmental defects without reliance on large-volume bone grafting. Following corticotomy, gradual distraction generates vascularised regenerate bone whilst a bone segment is transported across the defect [26–29]. Treatment comprises latency, distraction, and consolidation phases, with distraction conventionally undertaken at approximately 1 mm/day in three or four increments. Traditional bone transport uses circular or monolateral external fixation, whereas contemporary methods include plate- or nail-assisted transport and fully internal motorised systems. The transported segment is brought into contact with the opposite bone end at the docking site, where compression promotes union. Distraction at the corticotomy generates new bone to restore length; frame adjustments can also correct associated deformity [30–32].

Its principal advantages are the avoidance of large-volume bone grafting and the ability to address bone loss, shortening and deformity simultaneously. Distraction may also promote local vascular and inflammatory responses that support infection control, although infection eradication remains dependent on adequate debridement and appropriate antimicrobial therapy [33, 34].

Reported union rates approach 90%, although complications are common, treatment may be prolonged and substantial patient adherence is required [1, 28]. These include pin-tract infection, regenerate insufficiency, axial deviation, docking-site non-union, residual limb-length discrepancy, joint stiffness, contracture, and refracture. In this combined technique, a cement spacer first occupies the defect and induces a membrane. After spacer removal, the bone segment is transported through that membrane; this may improve the local biological environment, although comparative evidence remains l**i**mited [35].

### Bone grafting

Autologous bone grafting remains the gold standard for reconstruction of smaller critical-sized bone defects, providing osteogenic, osteoinductive, and osteoconductive properties [36]. Cancellous graft may be harvested from the iliac crest or using the reamer–irrigator–aspirator technique. Its use in larger defects is constrained by graft-volume requirements, resorption, limited intrinsic mechanical strength, and donor-site morbidity. Allograft and synthetic graft substitutes may increase volume but are principally osteoconductive and should generally be regarded as graft extenders rather than substitutes for autologous graft in biologically compromised defects [37].

### Segmental graft reconstruction

Structural cortical autografts or segmental allografts may restore length and provide immediate structural support in selected diaphyseal defects [38]. For example, tricortical iliac crest autografts have been used to reconstruct segmental defects of the radius and ulna [39]. Their incorporation depends on stable fixation, close host–graft contact, adequate vascularity, and durable soft-tissue coverage. Limited revascularisation of large structural grafts increases the risks of infection, non-union, resorption, fatigue fracture, and late mechanical failure. This approach is therefore best reserved for carefully selected, biologically favourable defects.

### Induced-membrane technique

The Masquelet induced-membrane technique is a two-stage procedure for critical-sized defects [22]. The first stage comprises radical debridement, stable fixation, insertion of an antibiotic-loaded polymethylmethacrylate spacer, and definitive soft-tissue reconstruction where required. The spacer maintains defect dimensions, manages dead space, permits local antibiotic delivery, and induces a vascularised membrane containing osteogenic and angiogenic mediators.

At the second stage, typically after 4–8 weeks, the membrane is carefully incised and preserved, the spacer removed, and the resulting cavity filled predominantly with autologous bone graft before membrane closure. As the graft provides little immediate mechanical stability, fixation must remain sufficiently stable throughout incorporation. Success depends on radical debridement, infection control, stable fixation, viable host-bone ends, adequate soft-tissue coverage, and preservation of the membrane. Reported union rates approach 85%, with favourable outcomes associated with antibiotic-loaded spacers, autologous graft, and stable intramedullary fixation [24, 25]. The influence of defect size remains uncertain, although increasing defect volume imposes greater biological and graft-volume demands.

### Free vascularised fibular grafting

A free vascularised fibular graft transfers living cortical bone with an intrinsic blood supply, providing both biological activity and structural continuity [18]. It may be considered for large defects or poorly vascularised recipient beds, particularly when conventional graft incorporation is unlikely. Double-barrel constructs or supplementary fixation may improve initial mechanical strength [40].

The technique requires microsurgical expertise and suitable recipient vessels, which may be compromised following high-energy trauma or infection. Hypertrophy sufficient for unrestricted loading may take considerable time, particularly in tibial reconstruction, and outcomes can be unpredictable. Complications include microvascular thrombosis, stress fracture, non-union, malalignment, infection, and donor-site morbidity [41]. Bone transport or induced-membrane reconstruction may therefore provide more predictable alternatives in appropriately selected patients.

### 3D printed implants

Patient-specific three-dimensional (3D)-printed implants are an emerging option for reconstructing critical-sized bone defects, particularly in complex diaphyseal and periarticular cases. These implants are commonly manufactured from polyetheretherketone (PEEK), although porous titanium and other biomaterials may be used. Advances in additive manufacturing have enabled patient-specific 3D-printed scaffolds with controlled architecture and improved biomechanical properties [42–44]. These constructs enable anatomical restoration of the defect and may be combined with internal fixation, arthrodesis, and bone grafting to provide structural support and promote osseointegration [31, 32].

Definitive implantation is generally preceded by an acute stage comprising debridement, temporary stabilisation, infection management, and soft-tissue reconstruction; it should therefore not be considered a true single-stage technique. Potential advantages include patient-specific reconstruction, immediate structural support, and avoidance of prolonged distraction osteogenesis [45]. Limitations include cost, manufacturing time, regulatory requirements, infection risk, and limited long-term clinical data.

### Amputation

Amputation may offer a more predictable route to rehabilitation and an acceptable functional outcome and should not be regarded as treatment failure. In selected patients, it may provide the most reliable and rapid route to rehabilitation. Indications include an inability to eradicate infection, irreversible musculotendinous loss or severe, irreversible nerve injury resulting in a non-functional limb, inadequate soft-tissue coverage, severe limb trauma, patient frailty or comorbidity that precludes prolonged reconstruction, or local tissue conditions that make successful healing unlikely**.** Amputation may also be appropriate where the burden and duration of reconstruction are disproportionate to the anticipated benefit. Bone loss alone is rarely an indication for amputation [46]. The long-term physical, psychological, and prosthetic implications must be considered and discussed with the patient.

Non-operative management has almost no role in a critical-sized defect because, by definition, the structural integrity of the skeleton is compromised [12]. It may be appropriate only when the patient is moribund or medically unable to undergo operative treatment.

### Future directions

Emerging technologies in the management of critical-sized bone defects focus on optimising the biological environment for bone regeneration. Contemporary bioceramic scaffolds are increasingly being engineered to provide not only structural support but also local delivery of antibiotics, growth factors and osteogenic cells, while modulating the immune response through promotion of pro-regenerative macrophage phenotypes [47]. Although these approaches have demonstrated promising preclinical results, clinical evidence remains limited and high costs constrain wider adoption**,** and established reconstructive techniques, including the Masquelet induced membrane technique, distraction osteogenesis and vascularised bone transfer, continue to represent the standard of care [48].

## Conclusion

CSDs remain a significant reconstructive challenge. Successful management requires meticulous debridement, stable fixation, optimisation of the soft-tissue envelope, and restoration of the biological environment for healing. Reconstructive strategy should be individualised according to defect characteristics, host factors, and surgical expertise. Emerging technologies, including patient-specific implants and tissue engineering, are promising but require further clinical validation.

## Data Availability

No datasets were generated or analysed during the current study.
